# Intralipid Infusion to Fetal Sheep ( *Ovis aries* ) Promotes Differentiation and Lipid Accumulation of Adipose Tissues

**DOI:** 10.1096/fj.202502000R

**Published:** 2025-10-29

**Authors:** Xinrui Li, Sarah M. Alaniz, Samantha Louey, Jeanene Marie Deavila, Sonnet S. Jonker, Min Du

**Affiliations:** ^1^ Nutrigenomics and Growth Biology Laboratory, Department of Animal Sciences Washington State University Pullman Washington USA; ^2^ Center for Developmental Health Oregon Health & Science University Portland Oregon USA

**Keywords:** adipogenesis, fetal sheep, inflammation, infusion, Intralipid, lipid accumulation

## Abstract

Intralipid is a lipid emulsion used for preterm infants, but its biological effects on adipose development remain poorly examined. We investigated the effects of intravenous Intralipid on the adipose tissue development of midgestation fetal sheep. Intralipid20 infusion was started in fetuses at 88–90 days of gestation (dG) following clinical infant dosing guidance (0.5–1 g/kg/d estimated body weight, increasing daily by 0.5–1 g/kg/d to a maximum of 3 g/kg/d). Intralipid increased perirenal fat weight, adipocyte size, and lipid accumulation. Expression of key adipogenic genes was upregulated, including *Pparg* (Peroxisome Proliferator‐Activated Receptor Gamma), *Fasn* (Fatty Acid Synthase), *Fabp4* (Adipocyte Fatty Acid Binding Protein 4), *Acca* (Acetyl‐CoA Carboxylase α), and *Cebpa* (CCAAT/Enhancer‐Binding Protein Alpha), while downregulating *Pdgfra* (Platelet‐Derived Growth Factor Receptor Alpha), *Cidea* (Cell death‐inducing DNA fragmentation factor α‐like effector A), *Ppargc1a* (Peroxisome Proliferator‐Activated Receptor Gamma Coactivator 1‐Alpha), and *Ucp1* (Uncoupling Protein 1). Similar changes were confirmed in omental fat. Intralipid elicited mild inflammation in both perirenal and omental fat by increasing the expression of *Tnfa* (Tumor Necrosis Factor‐Alpha), with upregulation of *Tlr4* (Toll‐Like Receptor‐4), and *Tlr2* (Toll‐Like Receptor‐2) specifically in perirenal fat. Notably, this inflammatory response occurred without elevation of *Il‐6* (Interleukin‐6) expression or NF‐κB activation. Moreover, Intralipid treatment suppressed the expression of fibrosis‐related genes, including *Lh2b* (lysyl oxidase‐like 2b) and *P4ha* (prolyl 4‐hydroxylase). These findings suggest that Intralipid administration at a developmental stage prior to normal term enhances ovine adipogenic differentiation and lipid accumulation which might be protective to premature infants, but it might slightly induce inflammation in the adipose tissues.

## Graphical abstract

Intralipid infusion during midgestation enhanced adipose development in fetal sheep by increasing fat mass, adipocyte size, and lipid accumulation. Expression of adipogenic genes (*Pparg*, *Cebpa*, *Fasn*, *Fabp4*, *Acca*) was upregulated. Intralipid elevated serum glucose, triglycerides and total cholesterol, induced mild inflammation via *TNFa*, *Tlr2*, and *Tlr4*. These findings highlight Intralipid's role in promoting adipogenesis and lipid storage while modestly activating inflammatory responses in developing adipose tissue.

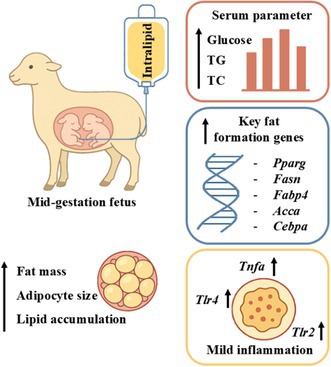

## 1 Introduction

Preterm infants which account for 10.4% of live births in the United States face unique nutritional challenges due to their incomplete tissue/organ development, limited fat reserves, high energy demands, and immature digestive systems [1]. These are closely related to the development of adipose tissue, which begins early in embryogenesis, including the commitment of mesenchymal stem cells to preadipocytes, followed by differentiation into mature adipocytes [2]. During normal circumstances, lipid levels in the fetal circulation are low [3], but they are essential for fetal brain development and adipose tissue formation at birth, as well as a variety of physiological functions [4]. As the gestational age progresses, the fetus gradually develops adipose tissue, providing the necessary energy reserves for after birth [5]. Due to their early departure from the intrauterine environment, premature infants miss the critical window of fetal fat accretion and are born with minimal adipose reserves, necessitating rapid postnatal fat accumulation to sustain growth and development [6]. To meet this need, premature infants often need special nutritional intervention, especially fat supplementation, which may lead to the exposure of premature infants to a high lipid concentration environment.

There are two main situations in which the fetus is exposed to a high lipid environment prematurely: One is maternal obesity or other metabolic diseases [7], and the other is premature birth that requires nutritional supplements [8]. In both cases, the fetus or premature infant will face unexpected lipid exposure. Pregnant women who are obese [9] or have hyperlipidemia [10] often exhibit dysregulated lipid metabolism, which can result in excessive accumulation of adipose tissue in the fetus. This abnormal fat deposition not only increases the risk of neonatal macrosomia but may also induce structural remodeling in fetal adipose tissue, cardiac tissue, and pancreatic islets [11, 12]. Premature infants often need intravenous nutrition support after birth due to their lack of enteral intake, including fat emulsion, which exposes them to a high‐fat state prematurely [8]. Since premature infants have not yet undergone a complete process of intrauterine growth and development of their adipose tissue [6], this artificial high‐fat exposure may affect the normal development of their adipose tissue. Therefore, a deep understanding of the development mechanism of adipose tissue is crucial to optimizing the nutritional management of premature infants.

To simulate the situation of premature infants being exposed to a high lipid environment at a critical developmental stage, we used a fetal sheep model administered Intralipid, a fat emulsion commonly used in total parenteral nutrition composed mainly of soybean oil, egg phospholipids, and glycerin [13]. We studied fetal sheep because of their developmental and allometric scaling similarities to humans, small litter size, and because of their tolerance to surgical intervention [14, 15, 16].

This study focused on two typical adipose tissues, perirenal fat and omental fat, which play important roles in metabolic regulation and their hypertrophy due to obesity is closely linked to metabolic dysfunction. We analyzed these two types of tissues, including changes in cell size and key molecular signaling pathways during the critical developmental window of the fetus. These findings are expected to provide an important theoretical basis for optimizing nutritional support strategies for premature infants and understanding the mechanism of action of lipid intervention on tissue development and metabolic programming.

## 2 Materials and Methods

### 2.1 Animals Care and Tissue Collection

A preterm sheep model was established through surgical intervention as previously described [17]. Briefly, pregnant ewes carrying twins underwent surgery at 84–86 dG (term is 147dG). Following surgery, the infusion commenced 4 days later (88–90 dG) and continued until 96–98 dG (Day 8). For each ewe, the two fetuses received different treatments: One fetus was administered a continuous intravenous infusion of Intralipid20 (0.5–1 g/kg/d estimated body weight, increasing daily by 0.5–1 g/kg/d to a maximum of 3 g/kg/d; *n* = 9), while the other fetus received Lactated Ringer's Solution as a control (*n* = 8). At the time of sample collection, perirenal fat and omental fat were harvested, with one portion embedded in OCT compound and frozen in liquid nitrogen‐cooled isopentane, while the other was wrapped in foil and frozen directly in liquid nitrogen. All samples were then stored at −80°C. Animal study and tissue collection were conducted at Oregon Health & Science University (IACUC protocol number 007).

### 2.2 Hematoxylin–Eosin (H&E) Staining

H&E staining of tissue was performed as described previously [18]. After bringing the cryosections to room temperature, they were fixed with 4% paraformaldehyde (PFA) for 30 min. Then, the sections were rinsed thoroughly with phosphate‐buffered saline (PBS) before proceeding with H&E staining. First, hematoxylin was used to stain the nuclei, followed by bluing in an alkaline solution. Then sections were stained with eosin. For long‐term preservation, the sections were dehydrated through a graded ethanol series (70%, 80%, 95%, and 100%), and xylene, and mounted with the cytoseal mounting medium. Finally, images were acquired using a Leica DM2000 microscope (Leica Microsystems Ltd., Germany). Adipocyte size was quantified using ImageJ software (National Institutes of Health, USA) by analyzing adipocytes with clearly defined and complete borders within each microscopic field.

### 2.3 Oil Red O Staining

Oil Red O staining was performed based on a published protocol [19]. The working solution was prepared by dissolving Oil Red O powder in isopropanol to a final concentration of 30%, then mixing it with double‐distilled water at a 3:2 ratio. The cryosections were brought to room temperature and fixed with 4% paraformaldehyde for 30 min. They were then incubated in 60% isopropanol for 10 min to prepare them for staining. The sections were immersed in the Oil Red O working solution for 30 min at room temperature, ensuring complete coverage. Finally, the sections were mounted with 90% glycerol, and images were acquired using a Leica microscope for visualization of lipid droplets. Quantification of Oil Red O‐stained areas was performed using ImageJ software (National Institutes of Health, USA), with results expressed as percentage area per microscopic field.

### 2.4 Measurement of Biochemical Parameters

Serum glucose concentration was measured using a calibrated glucose meter (Bayer Contour, Tarrytown, NY, USA). Measurement of serum biochemical parameters, including triglycerides (TG), high‐density lipoprotein cholesterol (HDL‐C), and low‐density lipoprotein cholesterol (LDL‐C), was performed according to the manufacturers' instructions using commercially available assay kits (Novus Biologicals, Centennial, CO, USA; catalog numbers NBP3‐24542, NBP3‐25823, and NBP3‐25881, respectively). Total cholesterol (TC) was calculated using Friedewald's equation: TC = LDL‐C + HDL‐C + (TG/2.2) [20].

### 2.5 Real‐Time Quantitative PCR (RT‐qPCR) Analysis

Sample tissue was ground in liquid nitrogen, homogenized in TRIzol Reagent (Ambion, #350303) using Polytron homogenization (Polytron PT 1200 E, Kinematica). After adding chloroform, the mixture was vortexed, incubated on ice and centrifuged at 12 000 *g*. The supernatant was precipitated with isopropanol, centrifuged and the RNA pellet was washed with 75% ethanol, air‐dried briefly, and resuspended in DEPC‐treated water. RNA concentration and purity were quantified using a NanoDrop spectrophotometer (Thermo Scientific, USA). cDNA synthesis was performed using the reverse transcription kit (Bio‐Rad, #1708891) to synthesize cDNA for further analysis. RT‐qPCR was conducted using Bio‐Rad SYBR Green (Bio‐Rad, #1725150) on the Bio‐Rad CFX system (Bio‐Rad, Hercules, CA, USA). The relative mRNA expression levels were quantified using the 2−^ΔΔCt^ method [21], with *Rpl13* as the reference gene, and the primer sequences are listed in Table 1.

**TABLE 1:** Primer sequences used for RT‐qPCR analyses.

Gene name	Sequence (5′‐3′)	
	Forward	Reverse
*Pparg*	ACGGGAAAGACGACAGACAAA	AAACTGACACCCCTGGAAGATG
*Fasn*	CTTAACAGCACGTCCCCCAT	TCCTCGGGCTTGTCTTGTTC
*Fabp4*	TCTCTCCCCAATTTGCAAC	TGATAACTTGGAAATAAGCCTA
*Acaca*	CCTGCCCGAGTTTTGAGTGG	ACTCTGGAGCGGATAAAACGG
*Cidea*	AAGGCCACCATGTACGAGAT	GGTGCCCATGTGGATAAGACA
*Ppargc1a*	CAGACCTGACACAACACGG	CTTGAAAAATTGCTTGCGTC
*Ucp1*	GAGTTCTTCACCACAGGGAAA	TCTGACCTTGACCACCTCTGT
*Tnfα*	ACACCATGAGCACCAAAAGC	AGGCACCAGCAACTTCTGGA
*Il‐6*	TCATCCTGAGAAGCCTTGAGA	TTTCTGACCAGAGGAGGGAAT
*Tlr4*	TGCTGGCTGCAAAAAGTATG	CCCTGTAGTGAAGGCAGAGC
*Tlr2*	CAAGAGGAAGCCCAGGAAG	TGGACCATGAGGTTCTCCA
*Lh2b*	ATGCCAATCAAGAGGATCTG	CAGGTAGCGTTTCCCAATGT
*P4ha*	GATAAGGCGCTTTTGCTCAC	ATCCACAGCAGCACCTTTTT
*Rpl13*	AGTACCGCTCCAAACTTATC	TCCTTCTTATAGACGTTCCG

### 2.6 Western Blot Analysis

Western blot analysis was conducted as described previously [22]. Tissue samples were homogenized in lysis buffer (50 mM Tris–HCl, pH 6.8, 2% SDS, 10% glycerol, and 0.01% Bromophenol Blue) using a Polytron homogenizer. The lysates were then boiled at 95°C for 5 min, aliquoted, and stored at −80°C for future analysis. Before gel loading, 5% β‐mercaptoethanol was added followed by reboiling. Proteins were separated using Bio‐Rad electrophoresis (80 V/30 min stacking, 120 V/90 min resolving) and transferred to nitrocellulose membranes (100 V/90 min). Membranes were blocked with 5% nonfat milk in TBST (1 h, RT), incubated with primary antibodies overnight at 4°C, then with secondary antibodies (1 h, RT). Protein bands were visualized using the LI‐COR imaging system (LI‐COR Biosciences, Lincoln, NE, USA), and band intensities were quantified using ImageJ (version 1.53c, National Institutes of Health, USA). Nuclear proteins were extracted using the NuCLEAR Extraction Kit (#NXTRACT, Sigma‐Aldrich) according to the manufacturer's instructions, and subsequent processing and analysis were performed as described above. Details of the antibodies used were listed: anti‐PPARγ (1:1000 dilution, #C26H11, Cell Signaling Technology, Danvers, MA), anti‐C/EBPα (1:1000 dilution, #D56F10, Cell Signaling Technology), anti‐UCP1 (1:1000 dilution, #D9D6X, Cell Signaling Technology), anti‐PGC‐1α (1:1000 dilution, #20658–1‐AP, Proteintech, Rosemont, IL), anti‐TNFα (1:1000 dilution, #AMC3012, Invitrogen), anti‐PDGFRα (1:1000 dilution, #5241, Cell Signaling Technology), anti‐p65 (1:1000 dilution, #8242, Cell Signaling Technology), antiphospho‐p65 (1:1000 dilution, #3033, Cell Signaling Technology), anti‐β‐tubulin (1:2000 dilution, #E7, Developmental Studies Hybridoma Bank, Iowa City, IA), anti‐Histone H3 (1:1000 dilution, #9715, Cell Signaling Technology), antimouse IRDye 680 (1:20 000 dilution, #C80926‐17, LI‐COR Inc., Lincoln, NE), and antirabbit IRDye 800CW (1:20 000 dilution, #C70918‐03, LI‐COR).

### 2.7 Statistical Analysis

Data were presented as mean ± SD. Statistical comparisons between groups were conducted using Student's *t*‐tests in GraphPad Prism version 8. Fetuses were considered independent experimental units for all analyses. Results were considered statistically significant at *p* < 0.05.

## 3 Results

### 3.1 Adipogenic Differentiation of Adipose Tissues in Fetal Sheep After Intralipid Treatment

**FIGURE 1:**
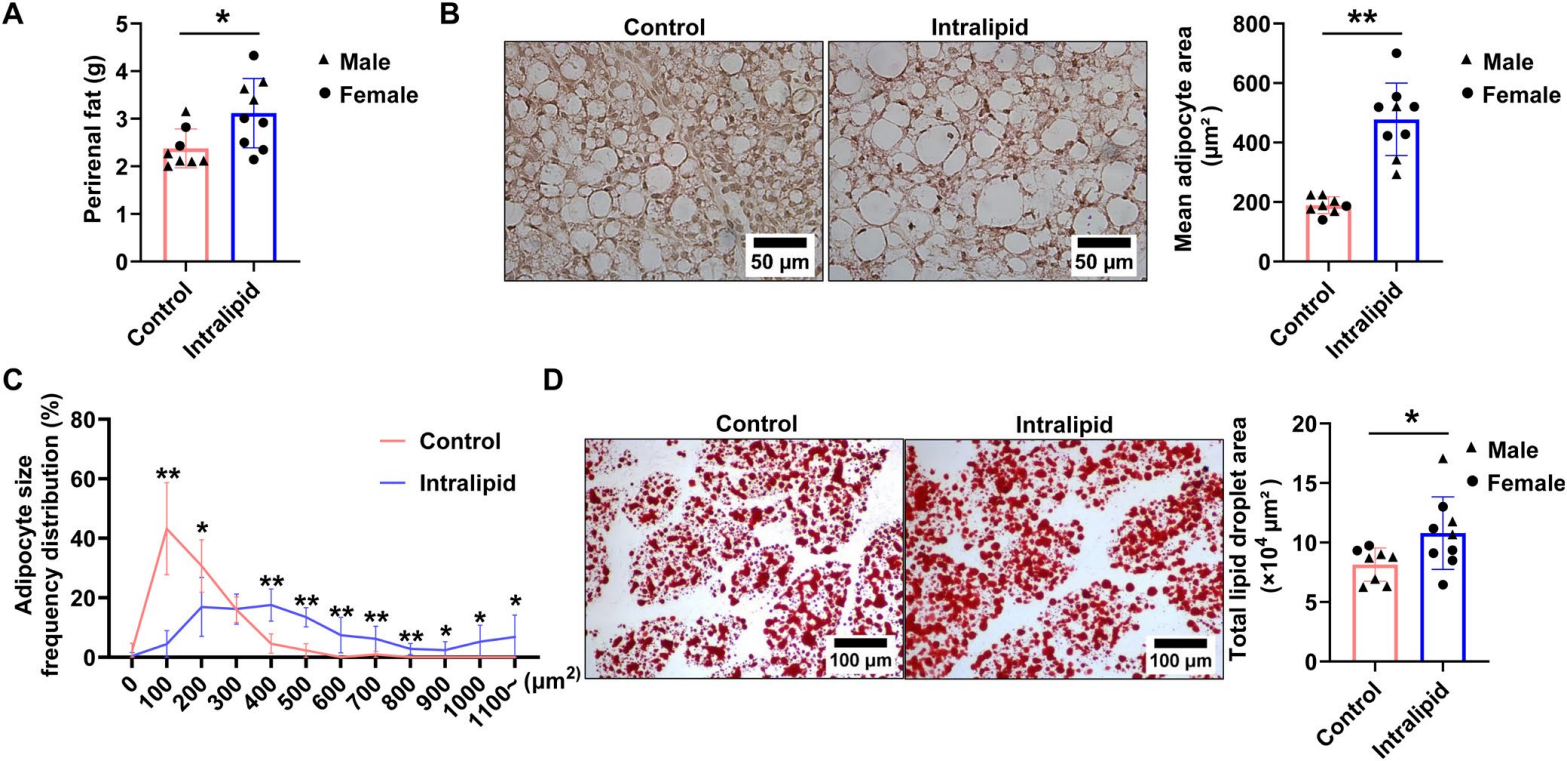
Effect of fetal Intralipid infusion on the structure of perirenal fat. (A) The weight of bilateral perirenal fat. (B) H&E staining of perirenal fat tissues (left) and mean adipocyte area (right); scale bars represent 50 μm. (C) Frequency distribution of adipocyte area. (D) Representative images of oil Red O staining for lipid accumulation (left) and quantification of total lipid droplet area (right); scale bars represent 100 μm. (Control group: *N* = 2F, 6M; Intralipid‐treated group: *N* = 6F, 3M; data are presented as mean ± SD; *p < 0.05, **p < 0.01).

**FIGURE 2:**
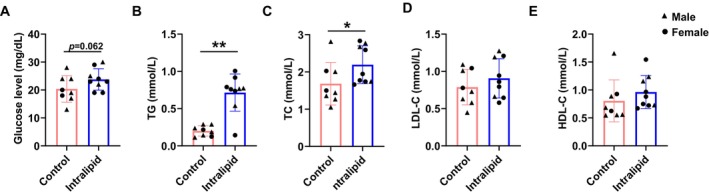
Effect of fetal Intralipid infusion on serum metabolic parameters. (A) Serum glucose concentrations. (B) Serum triglyceride (TG) concentrations. (C) Serum total cholesterol concentrations. (D) Serum low‐density lipoprotein cholesterol (LDL‐C) concentrations. (E) Serum high‐density cholesterol (HDL‐C) concentration. (Control group: *N* = 2F, 6M; Intralipid‐treated group: *N* = 6F, 3M; data are presented as mean ± SD; *p < 0.05, **p < 0.01).

**FIGURE 3:**
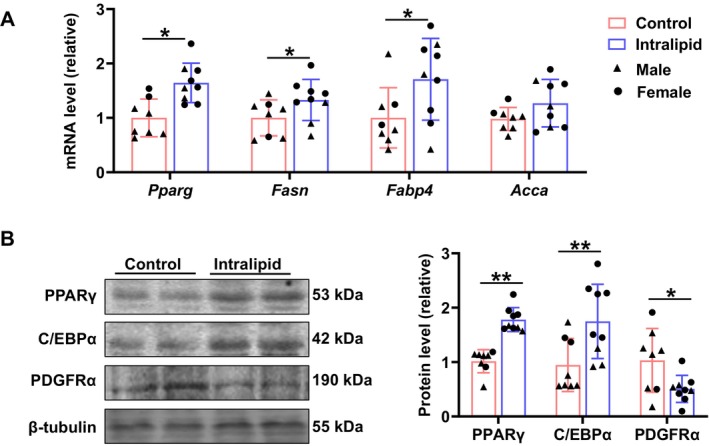
Fetal Intralipid infusion modulated perirenal fat development. (A) Relative mRNA expression levels of *Pparg*, *Fasn*, *Fabp4*, and *Acca*. (B) Protein levels of PPARγ, C/EBPα, and PDGFRα analyzed by Western blot. (Control group: *N* = 2F, 6M; Intralipid‐treated group: *N* = 6F, 3M; data are presented as mean ± SD; *p < 0.05, **p < 0.01).

As shown in Figure 1A, the perirenal fat mass of the Intralipid group was greater than that in the control group (*p* = 0.011). Parallelly, adipocyte sizes as assessed by H&E staining were larger in the Intralipid group (Figure 1B; *p* < 0.001) and adipocyte size distribution shifted to be larger in the Intralipid group (Figure 1C), suggesting an elevated adipogenic differentiation and enhanced lipid accumulation. Indeed, Oil Red O staining revealed the higher lipid accumulation in the Intralipid group compared with the control group (Figure 1D; *p* = 0.019). Intralipid infusion tended to increase serum glucose levels compared with the control group (Figure 2A; *p* = 0.062). Serum TG (Figure 2B; *p* = 0.003) and TC (Figure 2C; *p* = 0.035) concentrations were significantly elevated in the Intralipid group. In contrast, LDL‐C (Figure 2D) and high‐density lipoprotein cholesterol HDL‐C (Figure 2E) levels did not differ significantly between groups. Furthermore, as shown in Figure 3A, the mRNA expression levels of key adipogenic marker genes, *Pparg* (*p* = 0.001), *Fasn* (*p* = 0.038), and *Fabp4* (*p* = 0.022), were upregulated in the Intralipid group, while *Acca* expression (*p* = 0.054) showed a trend of upregulation. In agreement, protein abundances of PPARγ (*p* < 0.001) and C/EBPα (*p* = 0.005), markers of adipogenesis, were higher due to Intralipid infusion (Figure 3B). Correlated with accelerated adipogenesis, the Intralipid‐treated group had a lower protein level of PDGFRα (*p* = 0.041), a marker of progenitor cells compared with the controls (Figure 3B).

**FIGURE 4:**
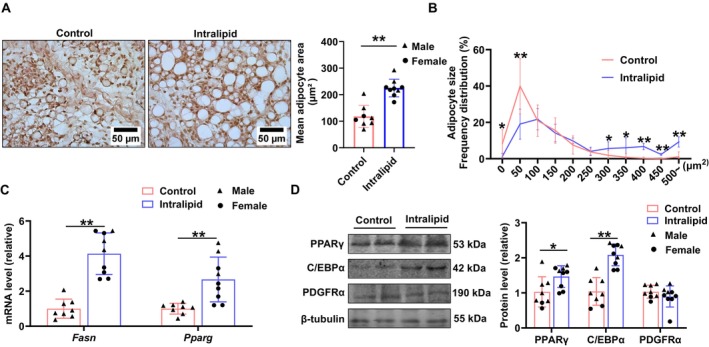
Fetal Intralipid infusion modulated omental fat development. (A) H&E staining of omental fat tissue (left) and mean adipocyte area (right); scale bars represent 50 μm. (B) Frequency distribution of adipocyte area. (C) Relative mRNA expression levels of *Fasn* and *Pparg*. (D) Protein levels of PPARγ, C/EBPα and PDGFRα detected by Western blot. (Control group: *N* = 2F, 6M; Intralipid‐treated group: *N* = 6F, 3M; data are presented as mean ± SD; *p < 0.05, **p < 0.01).

To further confirm fetal adipose tissue regulation by Intralipid exposure, we analyzed changes in the omental fat, another visceral fat with its hypertrophy closely associated with metabolic dysfunction [23]. As shown in Figure 4A, H&E staining revealed a larger adipocyte size in the Intralipid group (*p* < 0.001), and adipocyte size distribution shifted to be larger in the Intralipid group (Figure 4B). Similarly, mRNA expression levels of the adipogenesis‐related genes *Fasn* (*p* < 0.001) and *Pparg* (*p* = 0.001) were upregulated (Figure 4C). As shown in Figure 4D, the protein abundances of the adipogenesis markers, PPARγ (*p* = 0.041) and C/EBPα (*p* < 0.001) were substantially upregulated in response to Intralipid treatment, whereas PDGFRα levels remained unchanged. In combination, these data showed that Intralipid enhances adipogenic differentiation and lipid accumulation in adipose tissues of fetal sheep.

### 3.2 The Expression of Beiging Adipocyte Markers in Fetal Sheep due to Intralipid Infusion

**FIGURE 5:**
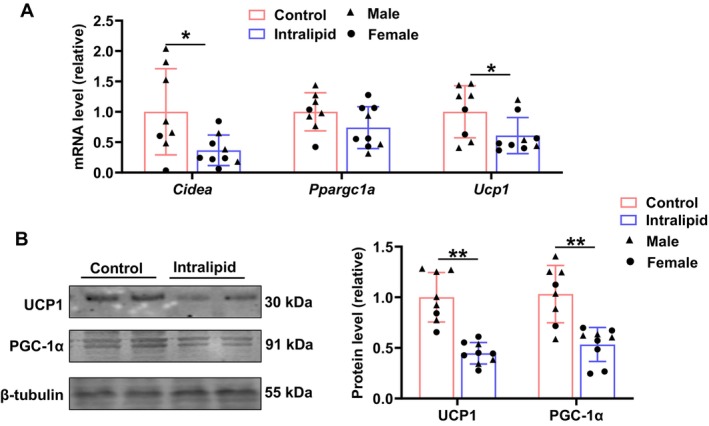
Fetal Intralipid treatment suppressed perirenal adipocyte beiging. (A) Relative mRNA expression of *Cidea*, *Ppargc1a*, and *Ucp1*. (B) Protein levels of UCP1 and PGC‐1α (The loading controls used in Figure 5B are identical to those used in Figure 3B because the experiments were done concurrently on the same samples). (Control group: *N* = 2F, 6M; Intralipid‐treated group: *N* = 6F, 3M; data are presented as mean ± SD; *p < 0.05, **p < 0.01).

Perirenal adipose tissue exhibits beiging [24], which refers to the adipocytes developing brown‐like characteristics and expressing thermogenic markers. The mRNA expression levels of key browning marker genes, *Cidea* (*p* = 0.011) and *Ucp1* (*p* = 0.021), were downregulated in the Intralipid group, while *Ppargc1a* expression (*p* = 0.063) showed a trend of downregulation (Figure 5A). Additionally, as shown in Figure 5B, UCP1 (*p* < 0.001) and PGC1‐α (*p* = 0.001) protein levels were markedly suppressed in response to Intralipid treatment. These results suggest that Intralipid infusion suppressed the beiging in the adipose tissue of premature sheep, potentially impairing thermogenic capacity.

### 3.3 Intralipid Induced an Inflammatory Response in Adipose Tissues of Fetal Sheep

**FIGURE 6:**
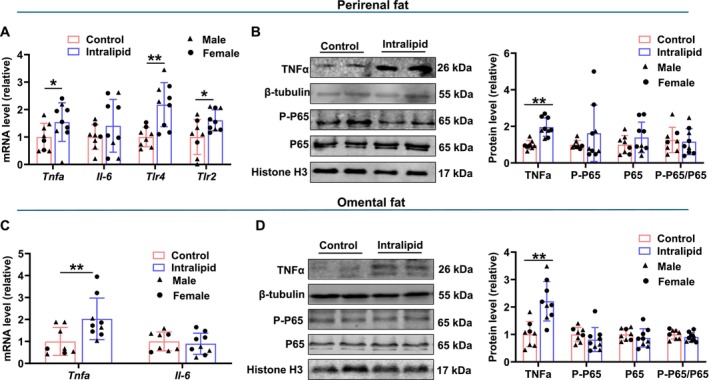
Inflammatory response of adipose tissues to fetal Intralipid infusion. (A) Relative mRNA expression of *Tnfa*, *Il‐6*, *Tlr4*, and *Tlr2* in perirenal fat. (B) Protein levels of TNFα, phosphorylated (P)‐P65 and total P65 in perirenal fat. (C) Relative mRNA expression of *Tnfa* and *Il‐6* in omental fat. (D) Protein levels of TNFα, P‐P65 and P65 in omental fat. (Control group: *N* = 2F, 6M; Intralipid‐treated group: *N* = 6F, 3M; data are presented as mean ± SD; *p < 0.05, **p < 0.01).

In the perirenal fat, as shown in Figure 6A, *Tnfα* mRNA expression was upregulated in the Intralipid group (*p* = 0.044) although *Il‐6* expression showed no difference. The mRNA levels of *Tlr4* (*p* < 0.001) and *Tlr2* (*p* = 0.014) were markedly higher in response to Intralipid treatment. Intralipid treatment also upregulated TNFα protein abundance (Figure 6B; *p* < 0.001). However, no differences were observed in the protein levels of total phosphorylated p65 (p‐p65) and p65 between the two groups (Figure 6B).

In omental fat, Intralipid treatment similarly led to a significant upregulation in *Tnfα* mRNA expression (Figure 6C; *p* = 0.001), while *Il‐6* expression remained unaltered (Figure 6C). Furthermore, the protein abundance of TNFα was elevated in response to Intralipid administration (Figure 6D; *p* = 0.001). Similar to perirenal adipose tissue, both phosphorylated and total p65 protein levels showed no differences in the omental fat (Figure 6D). Together, these findings indicate that Intralipid infusion might induce a mild proinflammatory response in midgestation adipose tissue.

### 3.4 Fibrosis in Adipose Tissue of Fetal Sheep Following Intralipid Treatment

**FIGURE 7:**
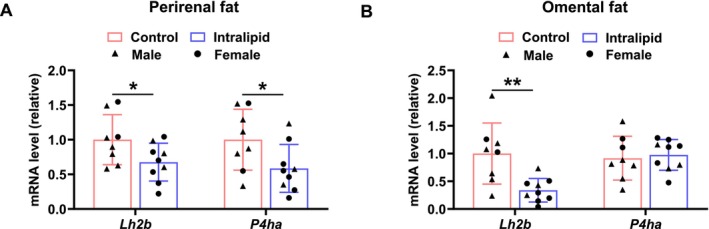
Fetal Intralipid infusion affects the fibrogenesis of adipose tissues. (A) Relative mRNA expression of *Lh2b* and *P4ha* in perirenal fat. (B) Relative mRNA expression of *Lh2b* and *P4ha* in omental fat. (Control group: *N* = 2F, 6M; Intralipid‐treated group: *N* = 6F, 3M; data are presented as mean ± SD; *p < 0.05, **p < 0.01).

Because progenitor cells in fetal adipose tissues have both adipogenic and fibrogenic capacities, we further analyzed the expression of fibrogenic‐related genes. Lysyl hydroxylase 2b (LH2b) and prolyl 4‐hydroxylase a (P4ha) are key enzymes regulating collagen cross‐linking [25]. In Figure 7A, Intralipid treatment inhibited the mRNA expression of *LH2b* (*p* = 0.026) and *P4ha* (*p* = 0.022) in perirenal fat. Similarly, as shown in Figure 7B, Intralipid treatment suppressed the mRNA expression of *LH2b* (*p* = 0.002), while having no significant effect on *P4ha* in omental fat. Taken together, these findings suggest that Intralipid treatment reduces the expression of key enzymes involved in collagen modification in adipose tissue, in consistency with the enhanced adipose tissue development of fetuses due to Intralipid infusion.

## 4 Discussion

In the current study, Intralipid was administered to midgestation fetal sheep for 8 days and we studied the effect on fetal adipose tissue. We found evidence of Intralipid‐stimulated adipogenesis and adipocyte enlargement, which resulted in increased fat mass. Additionally, there was a shift away from a beige phenotype, an inflammatory response in adipose tissue, and changed regulation of fibrogenesis. These differences may contribute to immediate adaptations in energy regulation, as well as predispose to obesity in later life [6].

Premature birth remains a major global health challenge, accounting for approximately 10% of all live births worldwide [26]. Due to insufficient time for development in the uterus, preterm infants are born with immature organ systems, particularly the underdeveloped digestive system and metabolic functions [27]. As a result, providing optimal nutritional support is essential for their growth, development, and overall survival. Intralipid not only provides energy, but also contains essential fatty acids, which play an important role in maintaining cell membrane integrity, promoting growth and development, and regulating inflammatory responses [28].

However, Intralipid can also cause unwanted effects. Studies also show that some preterm infants who received Intralipid infusion developed significant lipid accumulation in the lungs [29, 30]. The results of this study revealed significant lipid deposition in adipose tissue, consistent with previous reports showing that intravenous lipid emulsion therapy, has been documented to cause intravascular lipid deposition in vital organs including the liver [31], kidneys [30], and brain [29]. Moreover, literature reports indicated that acute high‐dose or rapid infusion of lipid emulsions can cause lipid overload in the circulation, potentially leading to fat microembolism, lipid accumulation in tissues, and subsequent organ dysfunction [32].

### 4.1 Effect of Intralipid on Adipogenesis and Lipid Accumulation

In this study, we assessed metabolic parameters in fetal sheep serum following Intralipid infusion. Our results demonstrated that Intralipid administration elevated serum glucose, TC, and triglyceride (TG) levels. These findings indicate that Intralipid not only directly promotes adipose tissue differentiation and lipid accumulation but also modulates systemic metabolic status. The observed elevations in serum glucose and lipid concentrations may reflect enhanced short‐term energy availability; however, they also imply a potential metabolic load [33]. These results are in line with previous reports showing that parenteral lipid emulsions can increase plasma lipid levels, potentially contributing to metabolic stress and promoting proinflammatory responses [34].

Our results indicate that Intralipid promotes adipogenesis and lipid accumulation in perirenal fat and omental fat. Mechanistically, Intralipid enhances adipogenesis primarily through the upregulation of key adipogenic transcription factors *Pparg* and *Cebpa*, and the resulting adipogenic markers *Fabp4*, *Fasn*, and *Acca*, along with reduced expression of *Pdgfra*. The downregulation of PDGFRα expression likely represents a cellular identity shift from progenitor cells to adipogenic differentiation [35], which is consistent with the subsequently observed increase in lipid accumulation and adipogenesis in the Intralipid treatment group. PPARγ, a key regulator of adipocyte differentiation in the nuclear receptor superfamily, promotes the differentiation of preadipocytes into mature adipocytes by elevating the expression of adipocyte‐specific genes [36, 37], including FABP4, FASN, and ACC, thereby facilitating lipid synthesis and storage [38, 39]. C/EBPα synergizes with PPARγ to regulate adipogenesis‐related gene expression [40, 41]. FASN is responsible for converting acetyl‐CoA and malonyl‐CoA into long‐chain fatty acids [42], with its upregulation generally associated with increased lipid accumulation [43]. FABP4 facilitates intracellular fatty acid transport and metabolism, influencing lipid handling and insulin sensitivity [44, 45, 46]. ACCα, the rate‐limiting enzyme in fatty acid biosynthesis, catalyzes acetyl‐CoA conversion to malonyl‐CoA, with its activity regulated by energy‐sensing pathways [47, 48]. Thus, the combined upregulation of these adipocyte‐specific genes indicates a clear activation of the adipogenic program under Intralipid stimulation. In this study, adipocyte‐specific genes were upregulated due to Intralipid, showing enhanced adipogenic differentiation and lipid accumulation, which should be beneficial for premature infants. Due to their shorter gestational age and lower lipid stores, they are at a higher risk of energy deficiency [49, 50]. Given their limited liver glycogen reserves, premature infants primarily rely on fat metabolism for energy [51, 52]. During late pregnancy, the fetus rapidly accumulates fat, which serves as a crucial energy reserve after birth [53]. In this study, Intralipid supplementation led to the accumulation of perirenal fat and omental fat droplets, which may serve as an essential energy source to support the growth and development of premature infants [54].

Excessive collagen deposition in adipose tissue, which restricts the hypertrophy of adipocytes and limits the ability of adipose tissue to expand [55], and triggers ectopic lipid deposition [56]. However, no detectable mRNA expression of Collagen Type I alpha 1 chain and Collagen Type III alpha 1 chain was observed in adipose tissue (data not shown), likely due to its early developmental stage. Nonetheless, Intralipid treatment resulted in a reduction in the mRNA expression of two key collagen‐modifying enzymes *Lh2b* and *P4ha*. The downregulation of *Lh2b* and *P4ha* may reduce collagen cross‐linking, leading to a looser extracellular matrix structure and loss of mechanical support for adipocytes [57, 58]. This alternation may enhance the plasticity of adipose tissue and thus improve their capacity to accumulate lipids.

### 4.2 Beige Adipose Tissue

The main function of beige adipose tissue in neonates is thermogenesis to prevent hypothermia, which also increases the energy demand of neonates [59]. In this study, the observed downregulation of thermogenic markers (CIDEA, PGC1‐α, and UCP1) in perirenal fat suggests impairment of thermogenesis. The reduced thermogenic capacity may significantly reduce caloric expenditure, redirecting energy toward storage rather than dissipation as heat. On the other hand, affected neonates may be more vulnerable to hypothermia and at higher risk for metabolic imbalances later in life [60].

### 4.3 Inflammation

Although the application of lipid emulsion injections can provide adequate energy, its other effects should also be evaluated. This study found that Intralipid might promote adipose tissue inflammation to some extent. TLR4 and TLR2 are important pattern recognition receptors in the innate immune system and play key roles in immune responses and inflammation [61]. Elevated circulating saturated fatty acids, function as endogenous ligands that directly engage TLR4 and TLR2 signaling complexes, thereby initiating proinflammatory cascades [62, 63]. Upon activation, TLR4 and TLR2 trigger downstream signaling pathways, including the NF‐κB pathway, which leads to the production of proinflammatory cytokines including TNF‐α and IL‐6 [64]. Our results showed that TNFα, a well‐established mediator of adipose inflammation [65], was significantly increased after Intralipid treatment, indicating a possible inflammatory response. However, no change in IL‐6 expression was observed, nor in the phosphorylation of P65, a key mediator of the classical NF‐κB pathway, was detected. The NF‐κB pathway is a central regulator of inflammation, and its activation typically leads to the transcription of additional proinflammatory genes, thereby sustaining and amplifying the inflammatory cascade [66]. The absence of P65 phosphorylation despite elevated TNF‐α levels suggests that the inflammatory response was not sufficient to activate this downstream pathway. Mechanistically, this partial inflammatory effect of fat emulsion might be related to its fatty acid composition. The ratio of n‐6 to n‐3 fatty acids in soybean oil emulsion is about 7:1. The n‐6 fatty acids can be elongated to arachidonic acid for the synthesis of proinflammatory eicosanoids, including prostaglandins, thromboxanes, and leukotrienes [67, 68]. Previous studies have shown that Intralipid infusion can amplify inflammatory responses by elevating plasma free fatty acid levels [69, 70]. Because the eicosanoids from n‐3 fatty acids such as eicosapentaenoic acid, demonstrate anti‐inflammatory effects, elevating the n‐3 fatty acid ratio in Intralipid may avoid the associated inflammation, which requires further studies.

## 5 Limitations

The relatively small sample size and unequal sex distribution prevented a robust sex‐specific analysis. Although individual data points with sex annotation have been presented for transparency, the findings should be interpreted with caution. Adipose tissue first emerges and progressively develops during midgestation in sheep, a critical window for adipogenesis [71]. Large‐scale studies indicate that at this developmental stage there are no significant sex differences in the timing or distribution of early fat deposition, suggesting that sex effects on adipose tissue development are not yet pronounced [72, 73]. While sex differences in adiposity typically become apparent during late gestation [74], these differences are unlikely to be detected at midgestation. Furthermore, the timing of our intervention, limited to a single midgestation point (~90 days), restricts the ability to fully capture the dynamic and stage‐dependent effects of Intralipid on fetal development. Adipose tissue ontogeny and metabolic programming are temporally regulated processes, with specific critical windows during gestation where nutritional and environmental factors differentially influence tissue growth, organ maturation, and long‐term health outcomes [75]. To comprehensively characterize the temporal trajectory of Intralipid's impact and translate these findings toward optimizing nutritional support in preterm infants, future research should include multiple gestational stages.

## 6 Conclusion

In conclusion, Intralipid supplementation promoted adipogenic differentiation and lipid accumulation in perirenal and omental adipose tissues of premature fetal sheep. Because adipose tissue deficiency is a significant concern in preterm infants, Intralipid infusion shows potential beneficial effects to support premature infant health by supporting postnatal energy availability. On the other hand, intravenous Intralipid injection might induce mild inflammation which warrants further studies.

## Author Contributions

**Xinrui Li:** investigation, data curation, formal analysis, visualization, writing – original draft. **Sarah M. Alaniz:** conceptualization, methodology. **Samantha Louey:** conceptualization, writing – review and editing. **Jeanene Marie Deavila:** investigation, formal analysis. **Sonnet S. Jonker:** conceptualization, supervision, writing – review and editing. **Min Du:** formal analysis, visualization, writing – review and editing, approval of final version.

## Conflicts of Interest

The authors declare no conflicts of interest.

## Data Availability

All data supporting the findings of this study are available from the corresponding author upon reasonable request.
